# Synthesis and Characterization of Dimmer-Acid-Based Nonisocyanate Polyurethane and Epoxy Resin Composite

**DOI:** 10.3390/polym9120649

**Published:** 2017-11-28

**Authors:** Xin He, Xiaoling Xu, Qian Wan, Guangxu Bo, Yunjun Yan

**Affiliations:** Key Laboratory of Molecular Biophysics of the Ministry of Education, College of Life Science and echnology, Huazhong University of Science and Technology, Wuhan 430074, China; n785888@163.com (X.H.); xuxiaoling@hust.edu.cn (X.X.); m201671694@hust.edu.cn (Q.W.); m201671171@hust.edu.cn (G.B.)

**Keywords:** hybrid nonisocyanate polyurethane, dimer acid, epoxy resin, coating

## Abstract

In this study, dimmer-acid-based hybrid nonisocyanate polyurethanes (HNIPUs) were synthesized by the one-step method without catalyst. Three polyamines and two epoxy resins were selected as raw materials for HNIPU, and cyclic carbonate was synthesized based on our previous work. All of the products were characterized by Fourier transform infrared spectroscopy (FTIR), differential scanning calorimetry (DSC), thermogravimetric analysis (TGA) and dynamic mechanical analysis (DMA). Then, HNIPU coatings were prepared and determined by swelling, water absorption, and water contact angle. The results showed that the HNIPU-4551 have the best mechanical and thermal properties because of its high crosslinking density. Among the different amines, it was confirmed that tetraethylenepentamine was the best amine curing agent for HNIPU coating. Meanwhile, the epoxy resin with a higher epoxy value would also form a higher crosslinking density. Those coatings showed an excellent impact strength, adhesion, flexibility, pencil hardness, hydrophilic, and appropriate crosslinking density.

## 1. Introduction

Polyurethanes (PUs) are one of the most popular polymer materials because of their versatile properties. They are widely used in thermoplastics, elastomers, foams, adhesives, coatings, textile, and packaging applications [1,2]. However, the synthesis process of PUs usually use diisocyanates, which can bring harmful and potentially carcinogenic components because of the instability and toxicity of the raw materials. Especially, the synthesis of diisocyanates need to use the phosgene, which are noxious substances [3,4].

When considering the evolution of national and international regulations, the synthesis research of nonisocyanate polyurethanes has been becoming an attractive area. The reactions of bis(cyclic carbonate)s with diamines yield urethanes with hydroxyl groups. The existence of hydroxyl groups allows for polymers to serve as reactive polyurethanes but limits their applications by their hydrophilicity. In other words, the secondary hydroxyl group forms hydrogen bonding with the urethane group which results in wonderful mechanical properties along with resistance to solvents and chemicals [5,6].

As one of the main raw materials for nonisocyanate polyurethanes, dimer acids (DAs) are green chemicals, which come from fatty acids by Diels-Alder reaction. DAs are promising biobased building blocks for the preparation of biobased polyamide (PA), PU, and other polymer materials [7,8]. Owing to their unique structures with long pendant chains, the synthetic polymers have specific properties, such as high flexibility and low glass transition temperatures.

In the nonisocyanate polyurethanes (NIPU) synthesis, different cyclic carbonates and diamines have been employed. Usually, cyclic carbonates are used as hard segment, and diamines are used as soft segment. However, isocyanates are used as hard segments, and active hydroxyl groups are used as soft segments in typical polyurethanes [9,10,11]. These segments provide polyurethanes with their characteristic exceptional versatility. It was reported that the nonisocyanatethat was synthesized from aromatic diamines showed a higher thermal stability than those from aliphatic amines, because of the stiffness of the aromatic chain [12].

Usually, NIPU has a low glass transition temperature and poor hardness [6,13]. Modification as a method to enhance performance had been studied for various fields. However, the modification for NIPU had very few reports. For example, Liu et al. used cyclic carbonate-functionalized polyhedral oligomeric silsesquioxanes to form NIPU/POSS coating [14]. Pathak et al. modified dehydrated castor oil fatty acid with *tri*-2-hydroxy-ethyl isocyanurate [6]. Therefore, epoxy resin with special chemical characteristics is an option that is worthy of consideration for modification. Because of the high adhesive strength, good impact resistance and high hardness, epoxy resin is widely used in modification applications [15]. For example, Rana et al. added antiplasticizers into the initial monomer mixture, and formed epoxy-amine thermosetting resins, leading to an improved toughness of the materials [16]. Bao et al. prepared nanocomposites with epoxy resin via in situ thermal polymerization [17].

**Scheme 1:**
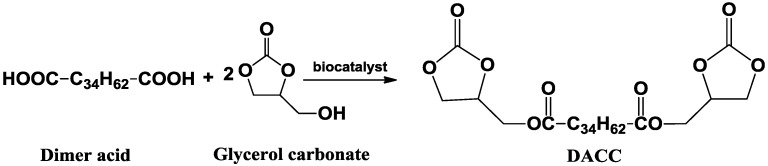
Synthesis of Dimer acid cyclic carbonate (DACC).

In the previous works, the dimer acid cyclic carbonate (DACC) was synthesized from glycerol carbonate (GC), and dimer acid (DA) using lipase as biocatalyst (Scheme 1). Then, the derivative was characterized and applied to synthesize nonisocyanate polyurethanes (NIPU) because of its environmental merits over the traditional polyurethanes. However, there is still large room to improve its thermal and mechanical properties in consideration of application requirement. Therefore, in this study, a simple modification method was further proposed. The dimer acid based hybrid nonisocyanate polyurethanes was synthesized with several amines and epoxy resins by one-step. Then, their thermal and mechanical properties were compared, and it was confirmed that the high secondary amine number and epoxy value would obtain a higher crosslinking density leading to better properties.

## 2. Materials and Methods

### 2.1. Materials

**Scheme 2:**
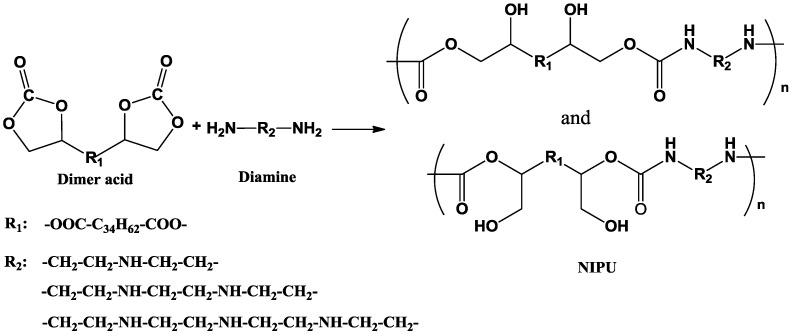
Synthesis of nonisocyanate polyurethanes (NIPUs).

Dimer acid cyclic carbonate (DACC) was synthesized based on our previous work (GC/DA molar ratio 8.00, reaction time 12 h, enzyme loading 8% (wt %, *w*/*w* DA), reaction temperature 50 °C, molecular sieves content 0.6 (wt, *w*/*w* DA), agitation speed 200 rpm and acetonitrile as the solvent) [18]. NIPU-23, NIPU-34, and NIPU-45 were, respectively, synthesized from DACC with dithylenetriamine, triethylenetetramine and tetraethylenepentamine (Scheme 2, solution polymerization, reaction time 12 h and reaction temperature 90 °C). Absolute ethyl alcohol, dichloromethane, dimethylsulfoxide (DMSO), diethylenetriamine (DETA), triethylenetetramine (TETA), and tetraethylenepentamine (TEPA) were of analytic reagents and were bought from Sinopharm Chemical Reagent Ltd., Co. (Shanghai, China). Bisphenol-A epoxy resin E-44 (mean epoxy value: 0.44) and Bisphenol-A epoxy resin E-51 (mean epoxy value: 0.51) were purchased from Yueyang Petrochemical Ltd., Co. (Yueyang, China). All of the materials were used as received without any further purification.

### 2.2. One-Step Method for HNIPUs and HNIPU Coatings

**Scheme 3:**
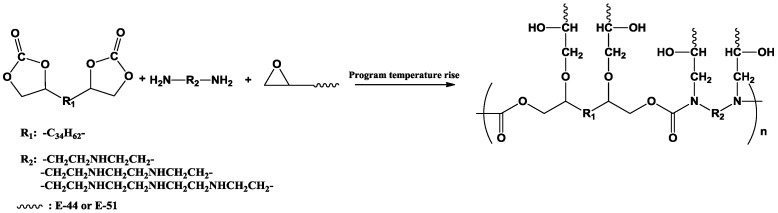
Synthesis of hybrid nonisocyanate polyurethanes (HNIPUs).

In this method, all HNIPUs were synthesized via a one-step, with solution polymerization of DACC, polyamines and epoxy resins (Scheme 3). First, DACC and polyamines were dissolved in dichloromethane with mechanical stirring for ten minutes. Second, absolute ethyl alcohol was added to the mixture, and epoxy resins were subsequently joined to the reaction. The reaction was carried out at a stoichiometric ratio. Before heating up, the mixture was poured into Teflon mold. The same synthetic method was also used in coating and the blending were cast on tin. The temperature of the reaction in the beginning was set at 40 °C and lasted for 3 h. Then, temperature was increased to 60 °C and was kept for 3 h. At last, the curing temperature of the mixture was set at 90 °C and remained for 6 h. After cooling to room temperature, the samples were kept in a desiccator for the future characterization. All of the raw materials of HNIPUs were summarized in Table 1.

### 2.3. Characterization

#### 2.3.1. Attenuated Total Reflectance (ATR)-Fourier transform infrared spectroscopy (FITR)

ATR-FTIR spectra were obtained using a Bruker Vertex 70 FTIR spectrometer (Karlsruhe, Germany) equipped with a DTGS detector and Attenuated Total Reflectance (ATR) accessory at room temperature. The FTIR spectra of the samples were recorded in the mid-infrared region (4000–400 cm^−1^) at 4 cm^−1^ resolution and 128 scans. All of the samples were only dried without other processing.

#### 2.3.2. Thermogravimetric Analysis

Thermogravimetric analysis (TGA) was conducted on a Pyris 1 TGA (Perkin-Elmer Instruments, Boston, MA, USA), under nitrogen at a heating rate of 10 °C/min from room temperature to 600 °C.

#### 2.3.3. Differential Scanning Calorimetry

Differential scanning calorimetry (DSC) was used to determine the glass transition temperature *T*_g_, where in samples were heated on a Perkin-Elmer Diamond DSC instrument (Boston, MA, USA) under N_2_ atmosphere from −50 °C to 100 °C at a heating rate of 10 °C/min.

#### 2.3.4. Dynamic Mechanical Analysis

The dynamic mechanical analysis (DMA) was performed on a Perkin-Elmer Diamond DMA instrument (Boston, MA, USA) under nitrogen atmosphere and single cantilever mode. The experiments were conducted for rectangular samples of the size 1 mm × 10 mm × 30 mm. The samples were subjected from −100 °C to 150 °C with a heating rate of 3 °C/min.

#### 2.3.5. Swelling Test

The swelling tests were carried out in DMSO as an appropriate solvent for the samples for 72 h at room temperature [19,20]. Before weighing, filter paper was used to absorb superficial solvent. The swelling was calculated by the following equation:

$$\mathrm{Swelling}\;\left(\%\right)=\frac{W_{t}-W_{o}}{W_{o}}\times 100$$

where *W*_o_ and *W*_t_ are the weight of samples before and after swelling, respectively.

#### 2.3.6. Water Absorption

All of the cured coating samples were tested by water absorption. The cured coating samples were taken from desiccator and then immersed in deionized water for 264 h at room temperature. The cured samples were removed from the deionized water, then cleaned by filter paper and weighed [21,22]. The water absorption was calculated by the following equation.

$$\mathrm{Water}\;\mathrm{absorption}\;\left(\%\right)=\frac{W_{a}-W_{o}}{W_{o}}\times 100$$

where *W*_a_ and *W*_o_ are the weight of samples after and before the water absorption, respectively.

#### 2.3.7. Water Contact Angle Test

The water contact angle of the different HNIPUs film surface was measured by an optical contact-angle measuring device (JC2000C1, Dataphysics Instruments Shanghai Zhongchen Digital Technic Apparatus Co., Ltd., Shanghai, China) at room temperature. Around 5 μL of water was automatically added by the instrument for each measurement. All of the measurements were conducted on static drops. Goniometry was employed in calculating the water angle by averaging the testing values of the repeated measurements.

#### 2.3.8. Coating Properties

The HNIPU coatings were further characterized for adhesion properties by cross cut test, as per Chinese National Standard (GB/T 9286-1998). A cross hatch cutter was used to cut a lattice pattern of cuts with equidistant spacing on the coating surface and commercial cellophane tape was applied over lattice. Pencil hardness of the coatings was measured on pencil hardness tester as per Chinese National Standard (GB/T 6739-2006). Flexibility and impact resistance of the coatings were tested by conical mandrel and impact tester as per Chinese National Standard (GB/T 1731-1993 and GB/T 1732-1993). Impact resistance was performed on the impact tester, with a maximum height of 50 cm and load of 1 kg.

## 3. Results and Discussion

### 3.1. ATR-FTIR

In this study, NIPU-23 (synthesized by DACC and DETA) and NIPU-2344 (composited with E-44 under one-step method) was selected to confirm the expected structure. Only NIPU-23 was chosen because the different NIPUs had no difference in the main functional groups, and NIPU-2344 was chosen by the same reason. Meanwhile, E-44 and E-51 have the same functional groups because they are both synthesized by epichlorohydrin and bisphenol A and just different in epoxy value [23,24]. So, only E-44 was selected as a contrast.

**Figure 1:**
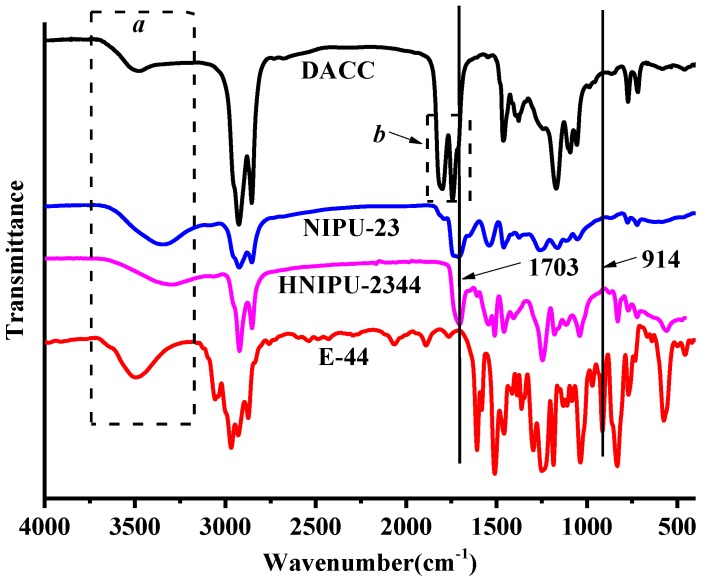
Fourier transform infrared spectroscopy (FTIR) spectra of synthesized HNIPU-2344-1 with their modification.

In the *a* region of Figure 1, DACC had few residual carboxyl groups, so the peak at 3400–3600 cm^−1^ was assigned to the hydroxyl group. E-44 also had a 3400–3600 cm^−1^ peak, because it contained hydroxyl groups that came from bisphenol A. For HNIPU-23 and NIPU-2344, the specific N–H stretching absorption of amide band at 3200–3400 can be observed clearly. In the *b* region, there were two peaks at 1802 and 1743 cm^−1^, which belonged to C=O cyclocarbonate group and C=O ester group, respectively. However, the C=O cyclocarbonate group was disappeared in NIPU-23 and HNIPU-2344. This indicated that the cyclocarbonate group had been opened by primary amine. The transmission at 1703 cm^−1^ of NIPU-23 and HNIPU-2344 was attributed to the urethane group and ester group. At last, when comparing HNIPU-2344 and E-44 at 914 cm^−1^, it was epoxide group and confirmed that the E-44 had completely reacted with amine [25].

### 3.2. Thermal Properties of HNIPUs

**Figure 2:**
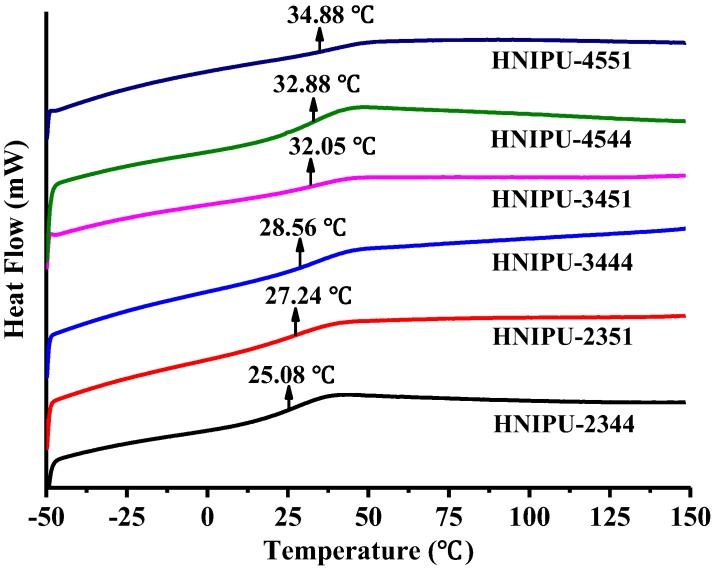
Differential scanning calorimetry (DSC) cures of the different HNIPUs.

The DSC curves and glass transitions (*T*_g_) data were presented in Figure 2 and Table 2. As reported in the literature, the crosslinking degree directly influences the value of *T*_g_ [26]. As seen in the Table 2, all *T*_g_ values of the HNIPUs were increased about 40 °C when compared with NIPUs, which indicates a high crosslinking degree of HNIPUs. It also infers that epoxy resin has crosslinked with secondary amine and formed a homogeneous crosslinking network. In addition, with the increase of secondary amine number, the *T*_g_ values order of HNIPUs was TEPA > TETA > DETA, due to the increases of crosslinking degree. Meanwhile, all of the HNIPUs composited with E-51 exhibited a higher *T*_g_ values than that of E-44. Therefore, high epoxy value plays an effective role in increasing the crosslinking degree, which significantly enhances the *T*_g_ of HNIPUs.

**Figure 3:**
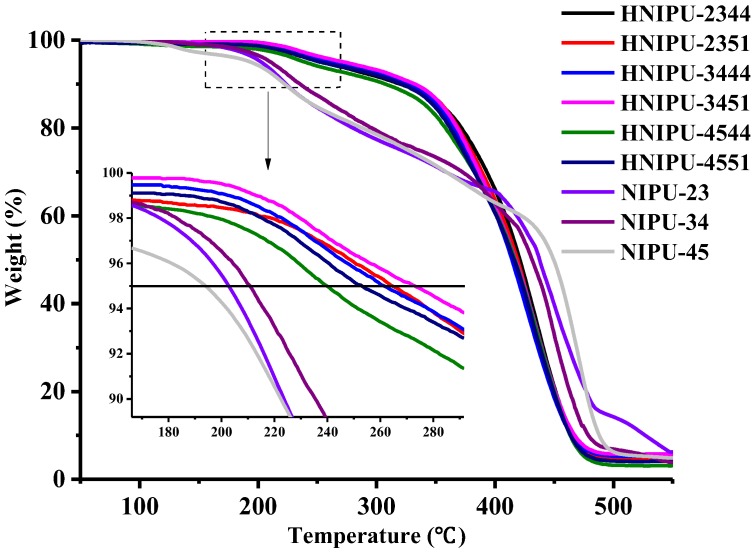
Dynamic mechanical analysis (TGA) cures of the different HIPUs and HNIPUs.

**Figure 4:**
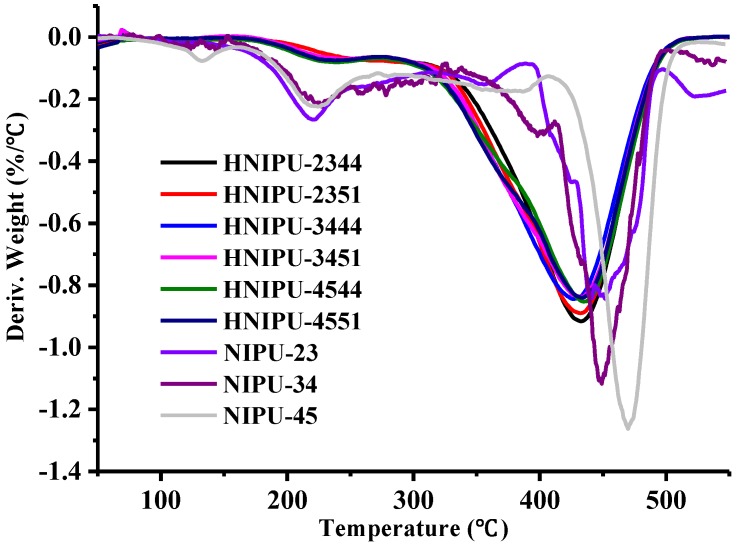
Differential thermal gravity (DTG) cures of the different HIPUs and HNIPUs.

The TGA was used to assess the thermal stability of the different HNIPUs under nitrogen atmosphere. The corresponding results were depicted in Table 2, Figure 3 and Figure 4, respectively.

In Table 2 and Figure 3, the results showed that all of the HNIPUs had a better thermal stability than NIPUs at 5% weight loss. When comparing the HNIPUs that are composited with E-44 and E-51, those from E-51 could bring a better thermal stability than from E-44 because E-51 had a higher epoxy value, which leads to a higher crosslinking degree. Interestingly, comparing the different diamines, the quality order of thermal stability was as follows: TETA > DETA > TEPA. This result was due to a better reactivity between the appropriate structures of TETA and cyclic carbonate [27]. HNIPU-3451 had the highest *Td*_5%_, about 62 °C increase in comparison with NIPU-34, which illustrates that epoxy resin and high reactive amine can obviously improve the thermal stability. As seen in Figure 3 and Figure 4, the *T*_max_ displayed in two steps, one for the degradation of soft segments, and another for the degradation of hard segments [28,29]. After combining with epoxy resin, the *T*_max_ in first step increased and a narrow distribution appeared, meanwhile, the *T*_max_ in the second step had a little decrease and exhibited a wider distribution, which confirms that epoxy resin can crosslink both with the soft segments and hard segments, leading to a good compatibility of HNIPUs. Consequently, the comprehensive thermal stability of HNIPUs was greatly enhanced.

### 3.3. Dynamic Mechanical Analysis

**Figure 5:**
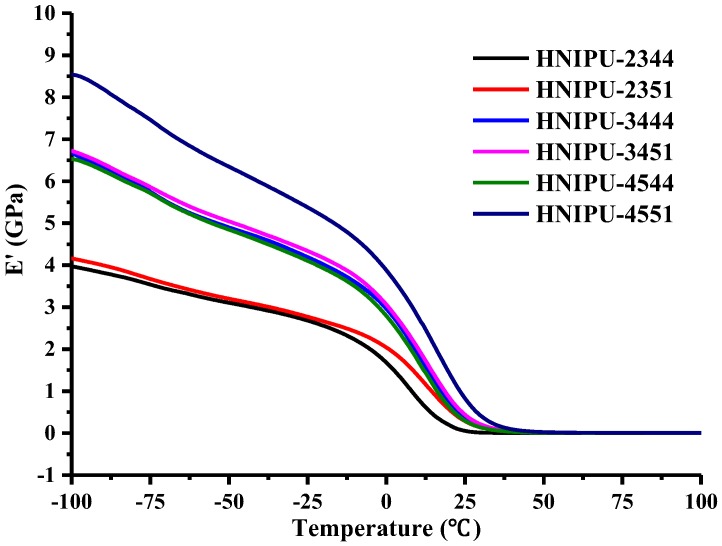
Storage modulus (*E′*) as a function of temperature for different HNIPUs.

**Figure 6:**
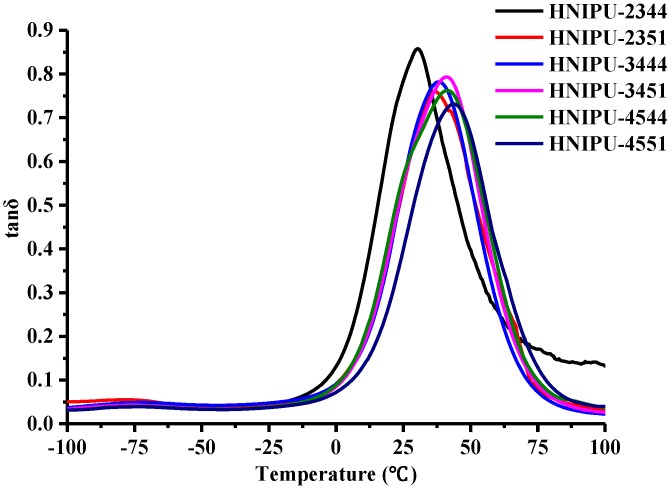
tanδ as a function of temperature for different HNIPUs.

DMA was used to analyze the thermomechanical properties of HNIPUs. Figure 5 and Figure 6, respectively, showed the temperature dependences of storage modulus (*E′*) and tanδ for different HNIPUs. The storage modulus (*E′*) represents the rigidity of the material, which reflects the crosslinking degree. Figure 5 indicated that HNIPU-4551 with the *E′* value of 8.55 GPa owned the highest storage modulus among all of the derived HNIPUs. The samples composited with E-51 possessed a higher storage modulus than those composited with E-44. Meanwhile, TEPA, with more secondary amine groups, could also lead to a higher storage modulus. The probable reason was that abundant epoxy groups and amino groups brought about a high crosslinking degree of HNIPUs, which strengthened their rigidity.

DMA also gave the glass transition temperature (*T*_g_) in Figure 6, and the data were listed in Table 2. HNIPU-4551 was observed with the highest *T*_g_ at 44.00 °C, but the *T*_g_ of HNIPU-2344 was only 29.70 °C. It was because the secondary amine groups would react with epoxy groups to form a high crosslinking density. Usually, the crosslinking density represented the concentration of cross-linked bonds per volume. Therefore, the higher the crosslinking density would lead to the stronger covalent bond inside polymer chains, the higher storage modulus and *T*_g_ [30,31,32]. The regularity of the results agreed with our DSC results in Figure 2.

### 3.4. Swelling Tests

**Figure 7:**
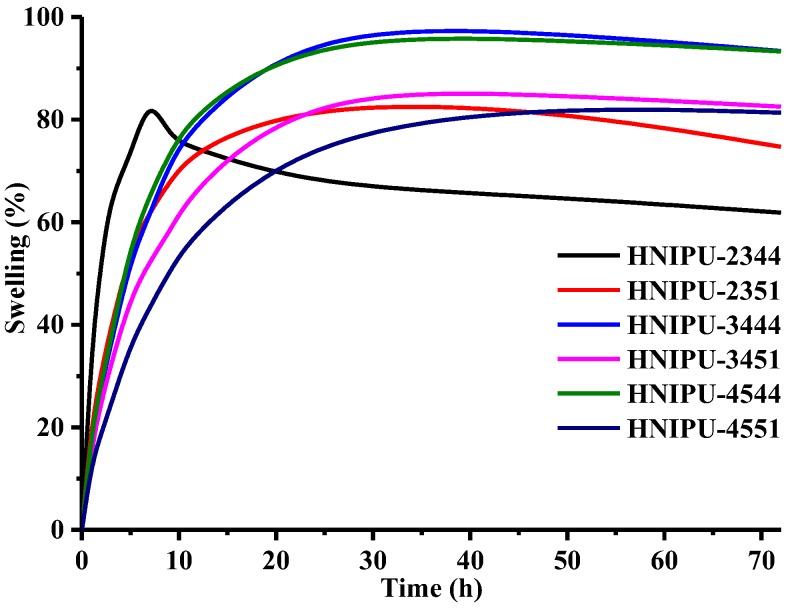
Swelling behavior of the different HNIPUs in dimethylsulfoxide (DMSO).

The swelling tests were used to investigate the strength of crosslinking degree about the samples by immersing them in solvent. DMSO was an appropriate solvent, which could dissolve the raw material, but not HNIPUs. As shown in Figure 7, the HNIPUs composited with E-44 showed a higher swelling than that composited with E-51. A strange phenomenon was that HNIPU-2344 and HNIPU-2351 were decreased after immersing for 8 h. The reason was probably that a large amount of DMSO immersed into the molecule interval and destroyed the network structures, which could not hold so much solvent any more. This phenomenon also suggested a low crosslinking degree of HNIPU-2344 and HNIPU-2351. When comparing different curves, it can be seen that the swelling of HNIPUs are influenced by both the number of epoxy groups and the secondary amine groups. However, the epoxy group number has a far greater impact on the crosslinking degree. The reason was that epoxy groups would react with themselves and hydroxyl groups to form a more stable structure.

### 3.5. Water Absorption

**Figure 8:**
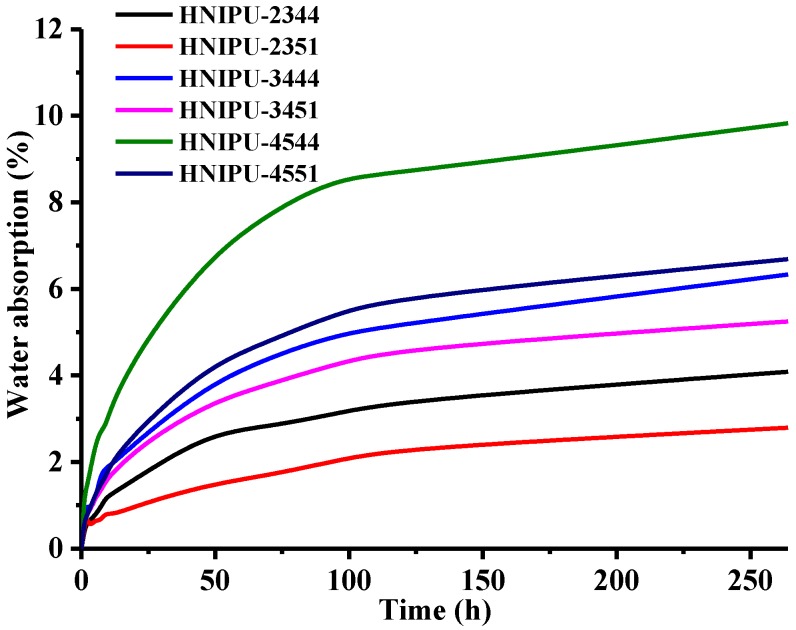
Water absorption behavior of different HNIPUs.

Generally, the free space and nature of the material influenced its water absorption behavior. The water absorption behavior of different HNIPUs was tested and presented in Figure 8. The HNIPUs composited with E-51 had a lower water absorption than that with E-44. The reason was that E-51 could provide a higher epoxy value to get a higher crosslinking. A high crosslinking density could hinder water molecules to pass through the HNIPU network structure [21,33]. In the meantime, owing to the hydrophobicity of urethane groups, the density of urethane groups could also influence the entry of water molecules. Therefore, the distance of the two primary amine groups would determine the water resistance. As the Figure 8 showed, the water absorption rank of HNIPUs was TEPA > TETA > DETA, which indicates that a higher density of urethane groups in the polymer chains can lead to a better hydrophobicity of HNIPU. Furthermore, the hydrogen bonding, Van der Waals force, and electrostatic interactions were existed between water molecules and unreacted secondary amine groups residual in the HNIPU. Consequently, the water absorption of HNIPU was influenced by crosslinking density, density of urethane groups, and intermolecular forces, especially by effects of the latter two.

### 3.6. Water Contact Angle

Table 3 showed the water contact angle of different HNIPUs films. HNIPU-2351 presented the highest contact angle of 87.25°. Meanwhile, all of the HNIPUs composited with E-51 were higher than those that were composited with E-44. Moreover, the contact angle increased with the increase of secondary amine group number. The lower water contact angle related to a greater wettability of the films, and the results were consistent with the water absorption tests.

### 3.7. Coating Properties

The HNIPU coatings were cured on mild steel and their mechanical properties were evaluated by pencil hardness, adhesion testing, impact strength, flexibility, and thickness. The long chain structure of dimer acid endowed HNIPU with a good flexibility. In Table 4, all of the HNIPU coatings showed excellent flexibility and arrived at 50 cm impact strength. In addition, adhesion was influenced and arrived the supreme grade of 0. Based on the reaction mechanism (Scheme 3), there were many extra hydroxyls in HNIPU, which led to a nice adhesion to mild steel. Pencil hardness of HNIPUs was increased with the increment of crosslinking degree. In addition, when comparing with epoxy value, the type of amines could significantly improve the pencil hardness. HNIPU-4551 showed the highest hardness, which was consistent with the data of DMA [6,34,35]. As a consequence, different amines and epoxy resins influenced the pencil hardness of HNIPUs and brought excellent adhesion, impact strength and flexibility.

## 4. Conclusions

Dimer-acid-based hybrid nonisocyanate polyurethanes were successfully synthesized and characterized. The type of amine and epoxy resin had a great role in the structure of HNIPUs. They could influence the crosslinking density to improve the thermal and mechanical properties. All of the results showed that a higher secondary amine number and epoxy group number could strengthen their properties, especially the hardness. In addition, the hydrophilic property was repressed by a density of urethane groups, i.e., a closer urethane group exhibited hydrophobic property. Different from traditional polyurethane modification, the NIPU composited with epoxy resin by one-step, and opening cyclic carbonate and compositing reacted together. The study confirmed that the epoxy value was more important for the crosslinking degree of HNIPU. Moreover, the HNIPU coatings showed nice properties, providing a good potential alternative for traditional polyurethane coating.

## Figures and Tables

**Table 1:** The raw materials of HNIPUs.

HNIPU Type	Amine Type	Epoxy Resin Type
HNIPU-2344	DETA	E-44
HNIPU-2351	DETA	E-51
HNIPU-3444	TETA	E-44
HNIPU-3451	TETA	E-51
HNIPU-4544	TEPA	E-44
HNIPU-4551	TEPA	E-51

**Table 2:** Thermal properties of the different nonisocyanate polyurethanes (NIPUs) and HNIPUs.

Samples	*Td*_5%_ (°C)	1st Step *T*_max_ (°C)	2nd Step *T*_max_ (°C)	*T*_g_ (°C)/DSC	*T*_g_ (°C)/DMA
NIPU-23	202.90	220.58	451.39	−14.64	–
HNIPU-2344	253.12	240.12	433.12	25.08	29.70
HNIPU-2351	263.98	240.98	431.98	27.24	36.30
NIPU-34	210.85	227.81	450.18	−9.23	–
HNIPU-3444	261.18	244.18	427.18	28.56	41.20
HNIPU-3451	272.74	244.74	432.74	32.05	37.70
NIPU-45	194.09	224.02	468.00	−6.00	–
HNIPU-4544	239.61	238.61	434.61	32.88	41.50
HNIPU-4551	252.57	237.57	433.57	34.88	44.00

**Table 3:** The water contact angle of different HNIPUs films.

HNIPU Films	Water Contact Angle (°)
HNIPU-2344	79.75 ± 1.75
HNIPU-2351	87.25 ± 2.25
HNIPU-3444	76.25 ± 1.25
HNIPU-3451	83.25 ± 1.75
HNIPU-4544	75.81 ± 2.69
HNIPU-4551	81.67 ± 2.33

**Table 4:** Mechanical properties of different HNIPU coatings.

	Pencil Hardness	Adhesion Testing	Impact Strength (cm)	Flexibility	Thickness (μm)
HNIPU-2344	B	0	50	7	442 ± 9
HNIPU-2351	HB	0	50	7	600 ± 10
HNIPU-3444	H	0	50	7	570 ± 17
HNIPU-3451	H	0	50	7	502 ± 17
HNIPU-4544	2H	0	50	7	471 ± 17
HNIPU-4551	3H	0	50	7	418 ± 12
